# Targeting the Sonic Hedgehog Signaling Pathway: Review of Smoothened and GLI Inhibitors

**DOI:** 10.3390/cancers8020022

**Published:** 2016-02-15

**Authors:** Tadas K. Rimkus, Richard L. Carpenter, Shadi Qasem, Michael Chan, Hui-Wen Lo

**Affiliations:** 1 Department of Cancer Biology, Wake Forest University School of Medicine, Winston-Salem, NC 27157, USA; TRIMKUS@wakehealth.edu (T.K.R.); rcarpent@wakehealth.edu (R.L.C.); 2 Department of Pathology, Wake Forest University School of Medicine, Winston-Salem, NC 27157, USA; sqasem@wakehealth.edu; 3 Comprehensive Cancer Center, Wake Forest University School of Medicine, Winston-Salem, NC 27157, USA; mchan@wakehealth.edu; 4 Department of Radiation Oncology, Wake Forest University School of Medicine, Winston-Salem, NC 27157, USA

**Keywords:** sonic hedgehog pathway, smoothened, GLI, tGLI1, inhibitors, PTCH, targeted therapy

## Abstract

The sonic hedgehog (Shh) signaling pathway is a major regulator of cell differentiation, cell proliferation, and tissue polarity. Aberrant activation of the Shh pathway has been shown in a variety of human cancers, including, basal cell carcinoma, malignant gliomas, medulloblastoma, leukemias, and cancers of the breast, lung, pancreas, and prostate. Tumorigenesis, tumor progression and therapeutic response have all been shown to be impacted by the Shh signaling pathway. Downstream effectors of the Shh pathway include smoothened (SMO) and glioma-associated oncogene homolog (GLI) family of zinc finger transcription factors. Both are regarded as important targets for cancer therapeutics. While most efforts have been devoted towards pharmacologically targeting SMO, developing GLI-targeted approach has its merit because of the fact that GLI proteins can be activated by both Shh ligand-dependent and -independent mechanisms. To date, two SMO inhibitors (LDE225/Sonidegib and GDC-0449/Vismodegib) have received FDA approval for treating basal cell carcinoma while many clinical trials are being conducted to evaluate the efficacy of this exciting class of targeted therapy in a variety of cancers. In this review, we provide an overview of the biology of the Shh pathway and then detail the current landscape of the Shh-SMO-GLI pathway inhibitors including those in preclinical studies and clinical trials.

## 1. Introduction

The Hedgehog (HH) gene was discovered in 1980 by Nusslein-Volhard and Wieschaus through genetic analysis of the fruit fly *Drosophila melanogaster* [1]. In the early 1990s, three HH gene homologs were discovered in vertebrates; Sonic Hedgehog (SHH), Indian Hedgehog (IHH), and Desert Hedgehog (DHH) [2,3,4]. DHH and IHH have been shown to play important roles in normal tissue development, including pancreas and testis organogenesis and bone formation [5,6,7,8]. Shh is the most potent of these ligands and is the most widely expressed in adult tissues [9,10]. Shh signaling plays an essential role in embryonic development and is critical for maintenance of tissue polarity. It has been shown that Shh is the dominant oncogenic HH ligand, as ectopic expression of Shh was sufficient to induce basal cell carcinoma in mice [11,12]. The Shh pathway is tightly regulated in most adult tissues but hyperactivation of this pathway is found in many solid tumors [13,14,15,16,17,18,19,20]. Aberrant Shh signaling has been implicated in many human cancers that account for up to 25% of human cancer deaths [21]. Greater understanding of the role of Shh signaling in human cancers has clearly indicated the need for development of anti-cancer therapies targeting the Shh pathway.

### 1.1. Shh Signaling Pathway Overview

**Figure 1:**
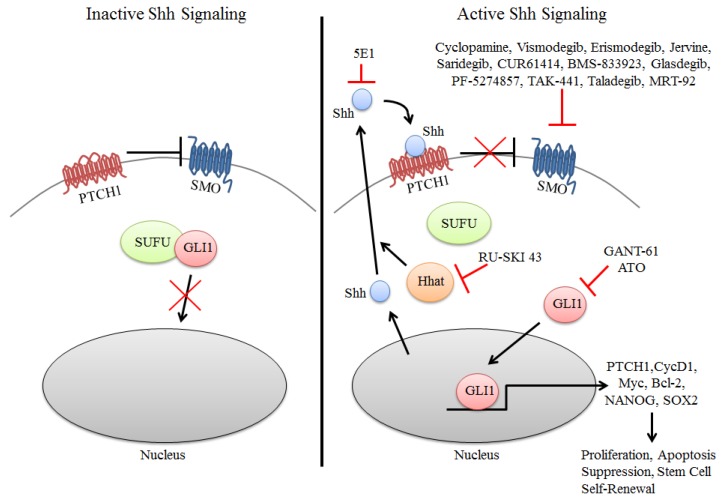
Inhibition of components of the Shh Pathway in cancer. Inactive signaling (left) occurs in the absence of Shh ligand wherein PTCH1 inhibits SMO resulting in GLI1 sequestration in the cytoplasm by SUFU. In the presence of Shh (right), PTCH1 suppression of SMO is abrogated resulting in the nuclear accumulation of GLI1 and activation of target genes that promote several oncogenic properties to tumor cells. Inhibition of the Shh pathway is primarily directed at inhibition of SMO and GLI1, with many of these compounds in clinical trials for solid cancers. More recently attempts have been made to inhibit the Shh signaling pathway by using the monoclonal antibody 5E1 or the SHHat inhibitor RU-SKI 43 to inhibit SHH directly. SHHat is an *O*-acyltransferase that catalyzes the palmitoylation of Shh, which is critical to its function.

The canonical HH pathway contains several key components, including HH glycoproteins Shh, IHH, and DHH [22]. Upon secretion, Shh glycoproteins bind and inactivate the 12-transmembrane protein Patched1 (PTCH1), which normally inhibits the activity of the 7-transmembrane protein Smoothened (SMO). In the presence of Shh ligand, PTCH1 inhibition of SMO at the primary cilium is abrogated resulting in the nuclear localization of glioma-associated (GLI) transcription factors, which are the terminal effectors of the Shh signaling (Figure 1). PTCH2 receptor shares approximately 54% homology with PTCH1, yet its expression pattern and signaling role in tissue vary significantly from PTCH1. PTCH2 is highly expressed in spermatocytes and helps mediate DHH activity in germ cell development [23]. It has also been shown that in the absence of Shh ligand binding, PTCH2 has a decreased ability to inhibit SMO [24]. In the absence of ligand, Suppressor of Fused (SUFU) negatively regulates the pathway by directly binding to GLI transcription factors and anchoring them in the cytoplasm preventing the activation of GLI target genes [25,26,27]. Cytoplasmic sequestration of GLI transcription factors by SUFU facilitates processing and degradation of GLI proteins, therefore inhibiting Shh pathway signaling [26]. SUFU has also been shown to form a repressor complex leading to interaction with DNA-bound GLI1 and suppression of GLI1-induced gene expression [28]. In vertebrates, there are three GLI transcription factors (GLI1, GLI12 and GLI3). GLI1 is the only full-length transcriptional activator whereas GLI2 and GLI3 act as either a positive or negative regulators as determined by posttranscriptional and posttranslational processing [29,30]. In response to Shh ligand binding, GLI2 accumulates in the primary cilium and drives transcriptional activation, overcoming negative regulation by GLI3 [31]. In addition to regulation by SUFU, GLI1 is also regulated by the kinase Dyrk1. Dyrk1 can potentiate GLI1 activity by phosphorylation at multiple serine/threonine sites that has been shown to induce nuclear accumulation and GLI1-mediated transcription [32]. GLI transcription factors can activate target genes that includes targets involved in HH pathway feedback (e.g., *GLI1*, *PTCH1*), proliferation (e.g., *Cyclin-D1*, *MYC*), apoptosis (e.g., *Bcl-2*), angiogenesis (e.g., *ANG1/2*), epithelial-to-mesenchymal transition (e.g., *SNAIL*), and stem cell self-renewal (e.g., *NANOG*, *SOX2*) [33,34,35].

In addition to the classical (canonical) signaling axis, there are also non-classical (non-canonical) pathways related to Shh signaling. Non-canonical Shh signaling refers to either: (1) activation of signaling from PTCH1/SMO but independent of GLI transcription factors; or (2) activation of GLI transcription factors independent of Shh ligand or PTCH1/SMO. The latter is better studied and multiple pathways have been identified, mostly oncogenic, that can increase GLI activity. GLI transcription factors have been shown to be positively regulated by K-Ras, TGF-β, PI3K-AKT, and PKC-α [36,37,38,39,40,41]. K-Ras, in particular, seems to be a pathway capable of activating GLI1 independent of the Shh pathway as knockdown of SUFU does not affect K-Ras-induced GLI1 [38,40]. Additionally, the GLI proteins have been shown to be negatively regulated by p53, PKA, and PKC-δ [42,43,44,45]. GLI1 transcriptional activity has also been shown to be reduced with p53 overexpression and enhanced with p53 knockdown [44]. Furthermore, p53 has been shown to interact with TAF9 leading to suppression of GLI1 activity [45]. PKA regulation of GLI1 is very specific as PKA directly phosphorylates Thr374 of GLI1, which promotes cytoplasmic localization and reduced activity of GLI1 [43].

### 1.2. Shh Signaling in Cancer

The role of dysregulated Shh signaling in cancer was first characterized by studies of basal cell nevus syndrome. Basal cell nevus syndrome, also known as Gorlin syndrome, is an autosomal dominant disorder that presents itself with craniofacial and skeletal abnormalities and a notably increased risk of advanced basal cell carcinoma and medulloblastoma [46,47]. The major breakthrough in the understanding of Shh signaling in cancer came from the discovery that mutations in *PTCH1* were the cause of Gorlin syndrome suggesting that aberrant Shh pathway activity was responsible for the development of these cancers [48,49]. These findings were reinforced by the discovery of mutations of *PTCH1*, *SMO*, and *SUFU* in a large percentage of spontaneous basal cell carcinomas and medulloblastomas [50,51]. The tumor suppressor role of PTCH1 has been further studied in transgenic mouse models that are heterozygous for a *PTCH1* null mutation. These mice showed the critical features of basal cell nevus syndrome, such as development of basal cell carcinomas, medulloblastomas, and rhabdomyosarcomas [48,49,52].

Abnormal Shh signaling is a hallmark of many cancers. It is now understood that somatic mutations in upstream pathway elements such as SMO and PTCH1 do not account for all of the dysregulated Shh signaling observed in tumors. It has been observed in multiple tumor types that Shh pathway dysregulation can also be induced in a ligand-dependent manner through enhanced Shh autocrine or paracrine signaling. This has been reported in a wide variety of cancers including pancreatic, colorectal, metastatic prostate carcinomas, and gliomas [19,53,54,55,56]. Furthermore, studies in epithelial cancers have found that tumor cells secrete Shh ligand in a paracrine fashion that stimulates production of secondary growth factors by stromal cells that drive tumor angiogenesis, tumor cell proliferation, and survival [57].

#### 1.2.1. Shh Signaling in Cancer Stem Cells

The Shh pathway has also been implicated in the regulation and maintenance of cancer stem cells (CSCs). In chronic myeloid leukemia and breast cancer, studies have found that Shh signaling is essential for maintenance of cancer stem cells and inhibition of the Shh pathway results in decreased stem cell propagation and renewal [14,58,59]. Recent studies have suggested that Shh signaling in CSCs in solid tumors is involved in metastatic progression and drives epithelial-mesenchymal transition of CSCs in pancreatic and colorectal cancers [56,60,61], providing a link between Shh signaling in regulation of normal stem cells and its role in cancer stem cell maintenance. 

#### 1.2.2. Novel GLI1 Isoform (tGLI1) in Cancer

Recently, a novel alternative splice variant of GLI1 was discovered in our laboratory, termed truncated GLI1 (tGLI1) [62]. tGLI1 is an alternatively spliced, shorter variant of GLI1 that contains an in-frame deletion of 41 amino acids corresponding to entire exon 3 and part of exon 4. Evidence to date indicates that tGLI1 is expressed at high levels in glioblastoma and breast cancer but undetectable or expressed at a low level in normal tissues. We have observed that tGLI1 promotes cell migration, invasion, and angiogenesis by upregulating CD24, HPA1, VEGF-C, and TEM-7 in glioblastoma and breast cancer [54,63,64]. We have also shown that tGLI1 upregulates VEGF-A and VEGFR-2, leading to a distinct autocrine loop that promotes angiogenesis and cell growth in breast cancer [65]. These studies have indicated tGLI1 is a gain-of-function transcription factor compared to GLI1 as it has shown the ability to upregulate several genes that are not GLI1 target genes. Overexpression of tGLI1 results in larger tumor growth and greater tumor angiogenesis compared to tumors overexpressing GLI1 [54,62,63,64]. We have observed tGLI1 expression in a high percentage of breast (78%) and glioblastoma (47%) tumors. Interestingly, tGLI1 is exclusively expressed in cancer cells and tissues as we have not detected tGLI1 in healthy cells or healthy human tissues [66]. Considering the potent oncogenic effects of tGLI1 and its unique expression pattern in cancer cells and tissues, tGLI1 is an optimal therapeutic target although no targeted inhibitors for tGli1 have been developed to date. The potential role tGLI1 plays in the lack of efficacy of inhibitors directed toward the Shh pathway, GLI1-targeted inhibitors in particular, has not been addressed.

Development of therapeutics for the canonical Shh signaling pathway has primarily focused on targeting SMO and GLI1. Natural and synthetic antagonists have been developed for both SMO and GLI1 with many having undergone clinical trials with varying degrees of success. SMO inhibition was first characterized through binding studies of cyclopamine, a natural steroidal alkaloid derived from *Veratrum californicum* [28,67,68]. Derivatives of cyclopamine have been developed in the hopes of increasing specificity and pharmacological potency. A setback in targeting SMO has been the observation of spontaneous mutations can develop as a response to some SMO inhibitors [69]. GLI1 has also been singled out as a therapeutic target as it is the most characterized GLI transcription factor associated with activation of Shh target genes although the library of GLI1 antagonists is not as extensive as for SMO. Here we review the current and rapidly expanding field of natural and synthetic SMO and GLI small-molecule inhibitors, attempting to provide a basis for future studies and development. We also highlight Shh pathway small-molecule inhibitors currently in preclinical studies and clinical trials.

## 2. SMO and GLI Inhibitors

### 2.1. SMO Inhibitors

SMO has been the primary target for the development of Shh-pathway inhibitors. SMO inhibition prevents the downstream activation of GLI transcription factors, leading to suppression of those genes associated with cancer growth and progression. Table 1 indicates the compounds targeting components with the Shh pathway and the clinical status of these compounds.

**Table 1:** Small molecule Shh pathway inhibitors in active clinical trials as of January 2016. Data sourced from www.clinicaltrials.gov.

Compound	Organization	Target	Cancer Type	Clinical Trial	NCT Trial
GDC-0449 (Vismodegib/Erivedge)	Roche/Genentech/Curis	SMO	Basal Cell Carcinoma - - - - - - - Advanced/Metastatic Basal Cell Carcinoma Basal Cell Nevus Syndrome Medulloblastoma - Recurrent Medulloblastoma Metastatic Castration-Resistant Prostate Cancer Chondrosarcoma Advanced Pancreatic Cancer Metastatic Pancreatic Cancer Myelofibrosis Metastatic Gastric & Esophageal Cancer Advanced Prostate Adenocarcinoma Small-Cell Lung Cancer Keratocystic Odontogenic Tumor Advanced Solid Tumors Acute Myeloid Leukemia Intracranial Meningioma	Phase II	NCT01835626
				Phase I	NCT02639117
				Phase II	NCT02067104
				Phase 0	NCT01631331
				Phase II	NCT01815840
				Phase II	NCT01700049
				Phase II	NCT01898598
				Phase IV	NCT02436408
				Phase II	NCT01367665
				-	-
				Phase II	NCT01556009
				Phase II	NCT00957229
				Phase I/II	NCT01601184
				Phase II	NCT01878617
				Phase II	NCT01239316
				Phase II	NCT00939484
				Phase 0	NCT02115828
				-	-
				-	-
				Phase II	NCT01267955
				Phase II	NCT01195415
				-	-
				Phase I	NCT00878163
				Phase II	NCT01088815
				Phase I	NCT02593760
				Phase II	NCT00982592
				-	-
				Phase I/II	NCT01163084
				-	-
				Phase II	NCT00887159
				Phase II	NCT02366312
				-	-
				Phase II	NCT02091141
				Phase II	NCT02073838
				Phase II	NCT02523014
LDE225 (Erismodegib/Sonidegib/Odomzo^®^)	Novartis	SMO	Prostate Cancer Castration-Resistant Prostate Cancer Pancreatic Adenocarcinoma Advanced/Metastatic Pancreatic Cancer Refractory Multiple Myeloma Recurrent Ovarian Cancer Triple-Negative Breast Cancer Myeloid Malignancies Basal Cell Carcinoma Advanced/Metastatic Basal Cell Carcinoma - Advanced Solid Tumors -	Phase I	NCT02111187
				Phase I	NCT02182622
				-	-
				Phase I/II	NCT01431794
				-	-
				Phase I/II	NCT02358161
				-	-
				Phase II	NCT02086552
				-	-
				Phase I/II	NCT02195973
				Phase I	NCT02027376
				-	-
				Phase I	NCT02129101
				Phase II	NCT00961896
				Phase II	NCT00961896
				Phase 0	NCT02303041
				Phase II	NCT01327053
				Phase I	NCT01769768
				Phase I	NCT01954355
LDE225 (Erismodegib/Sonidegib/Odomzo^®^)	Novartis	SMO	Advanced Gastroesophageal Adenocarcinoma Small Cell Lung Cancer Myelofibrosis Advanced/Metastatic Hepatocellular Carcinoma Relapsed Medulloblastoma	Phase I	NCT02138929
				-	-
				-	-
				Phase I	NCT01579929
				Phase I/II	NCT01787552
				Phase I	NCT02151864
				-	-
				-	-
				Phase II	NCT01708174
				-	-
BMS-833923/XL139	Bristol Myers Squibb/Exelixis	SMO	Basal Cell Nevus Syndrome Chronic Myeloid Leukemia	Phase I	NCT02100371
				-	-
				Phase II	NCT01357655
				-	-
PF-04449913 (Glasdegib)	Pfizer	SMO	Myelofibrosis Chronic Myelomonocytic Leukemia Myelodysplastic Syndrome	Phase II	NCT02226172
				Phase II	NCT01842646
				-	-
				-	-
				Phase II	NCT01842646
				-	-
LY2940680 (Taladegib)	Ignyta	SMO	Esophageal Cancer Advanced Solid Tumors	Phase I/II	NCT02530437
				Phase I	NCT01919398
				-	-
IPI-926 (Saridegib)	Infinity	SMO	Advanced Pancreatic Adenocarcinoma	Phase I	NCT01383538
				-	-
Arsenic Trioxide (ATO)	-	GLI1	Non-Small-Cell Lung Cancer Small Cell Lung Cancer Acute Myeloid Leukemia Hepatocellular Carcinoma - Malignant Glioma Myelofibrosis Acute Promyelocytic Leukemia - - - - - - - - Chronic Myelogenous Leukemia Acute Myeloid Leukemia Myelodysplastic Syndrome Chronic Myelomonocytic Leukemia	Phase I	NCT02066870
				-	-
				Phase II	NCT01470248
				Phase II	NCT01835288
				-	-
				-	-
				-	-
				Phase II	NCT02018757
				Phase I/II	NCT00275067
				Phase I	NCT01014546
				Phase III	NCT02339740
				Phase II	NCT01404949
				Phase II	NCT01409161
				Phase III	NCT00378365
				Phase IV	NCT01987297
				Phase III	NCT00866918
				Phase II	NCT00413166
				Phase II	NCT00551460
				Phase III	NCT00482833
				Phase IV	NCT02200978
				Phase I	NCT01397734
				-	-
				Phase II	NCT02188706
				Phase II	NCT02190695
				Phase II	NCT02188706
				Phase II	NCT02190695
				Phase II	NCT02190695
				-	-
				-	-

#### 2.1.1. Cyclopamine

Cyclopamine is an alkaloid isolated from *V. californicum* that showed strong potential to bind to SMO and inhibit the Shh signaling pathway [67,68]. Binding studies using a fluorescent cyclopamine derivative suggested that cyclopamine binds to the heptahelical transmembrane domain of SMO, preventing the necessary conformation shift to activate SMO [70,71]. Cyclopamine has been shown to inhibit tumor growth and proliferation in many mouse xenograft models, including human orthotopic glioma, melanoma, colon, pancreatic, and prostate cancers [55,56,60,72,73]. Although it significantly reduced tumor growth *in vivo*, cyclopamine never reached its therapeutic potential, as it caused many potent side effects, including weight loss, dehydration, and death in mouse models [74,75]. Several cyclopamine derivatives were developed to overcome solubility and stability issues and showed promise in *in vitro* studies, though *in vivo* evaluation of these compounds has not been conclusive [76,77,78].

#### 2.1.2. GDC-0449 (Vismodegib/Erivedge)

Vismodegib is a second generation cyclopamine derivative created by Roche/Genentech/Curis that binds directly to SMO to prevent GLI activation [79]. It was approved by the FDA in January 2012 as the first Shh pathway drug approved for treating any cancer. Vismodegib is currently being used to treat adults with metastatic basal cell carcinoma (BCC), or patients with recurrent, locally advanced BCC who are not candidates for surgery or radiation therapy [80]. In a subsequent clinical trial for metastatic BCC, vismodegib treatment resulted in tumor regression; however, after three months, a novel SMO mutation was discovered in the tumor tissue and the treatment progress ceased [81,82]. Recently, several other novel SMO mutations have been uncovered that may also play a role in vismodegib resistance [83]. Tumor stroma interactions may also play a role in the ineffectiveness of Shh pathway inhibition. Preclinical data showed that SMO inhibition could deplete the tumor stroma; however, a Phase Ib/II trial comparing gemcitabine in combination with vismodegib to gemcitabine with placebo for pancreatic ductal adenocarcinoma (PDAC) was terminated early because it did not meet the criteria for futility [84,85]. A follow-up study showed that while SMO inhibition in a genetic mouse model of PDAC resulted in decreased tumor stroma, the tumor itself was more aggressive, more highly vascularized, and more poorly differentiated, resulting in lower survival rates than controls [86]. It was later shown that the preclinical data for the original vismodegib trial was generated from mice that had developed large PDAC tumors, which did not allow for evaluation of long-term treatment effects, due to the short survival time of the mice [85]. Currently, vismodegib, as a monotherapy and in combination with other chemotherapeutics, is being studied in a long list of clinical trials in a wide array of cancers, including medulloblastoma, small cell lung cancer, metastatic pancreatic cancer, metastatic prostate cancer, intracranial meningioma, recurrent glioblastoma, and acute myeloid leukemia (Table 1).

#### 2.1.3. LDE-225 (Erismodegib/Sonidegib/Odomzo)

Erismodegib (also known as sonidegib and Odomzo^®^, East Hanover, NJ, USA) is an orally bioavailable SMO antagonist created by Novartis that has been shown to induce cell cycle arrest and apoptosis in a variety of cancer cell lines [87]. It has been effective in decreasing the epithelial-mesenchymal transition and invasive potential of several cancer types, including glioblastoma, prostate cancer, and renal cell carcinoma, implying that it can affect both tumor epithelial cells and cancer stem cells [88,89,90]. In July 2015, it was approved by the FDA to treat adult patients with locally advanced BCC that has recurred following surgery or radiation therapy, or those who are not candidates for surgery or radiation therapy. Upon this approval, LDE-225 became the second HH pathway inhibitor receiving FDA approval to treat cancer patients. Several Phase I/II trial for erismodegib as a monotherapy and in combination are underway, treating both solid tumors and hematological malignancies (Table 1).

#### 2.1.4. IPI-926 (Saridegib)

Saridegib came about as a result of chemical modification of the base structure of cyclopamine, and was determined to have inhibitory activity on the HH signaling pathway by its ability to suppress tumor growth of B837Tx medulloblastoma allografts in mice. Daily administration of 40 mg/kg led to tumor regression, with no recurrence after 30 days of treatment [91]. Saridegib has also been shown to decrease tumor growth in lung and chondrosarcoma xenografts [92,93]. Interestingly, saridegib did not affect epithelial tumor cells; however, when co-administered with gemcitabine in a mouse model of pancreatic ductal carcinoma, a marked increase in gemcitabine tumor delivery was recorded as a result of increased angiogenesis [85]. One Phase I clinical trial with saridegib is currently underway (Table 1).

#### 2.1.5. CUR61414

High throughput screening of over 100,000 compounds on a GLI-luciferase reporter assay in mouse C3H10T1/2 cells yielded CUR6414. Competition studies with cyclopamine and an SMO agonist showed that CUR6414 directly binds to SMO and prevents its activation [94]. In a PTCH^+/−^ mouse embryonic BCC model, CUR6414 was able to prevent the formation and promote the regression of spontaneous and UV-induced basaloid lesions without affecting normal basal keratinocytes. The lesions also showed increased tumor cell apoptosis [95]. A Phase I clinical trial for treatment of basal-cell carcinomas was stopped due to the failure of the drug to penetrate human skin [96].

#### 2.1.6. BMS-833923/XL139

BMS-833923, a Bristol-Myers Squibb/Exelexis product, was discovered to bind SMO by competition studies against a fluorescent-tagged cyclopamine, BODPIY-cyclopamine. It decreased *GLI1* and *PTCH1* mRNA expression *in vitro* and inhibited proliferation of several cancer cell lines [97,98,99]. *In vivo* studies showed that BMS-833923 reduced medulloblastoma, pancreatic carcinoma, and human cholangiocarcinoma xenograft growth in mice [97,98]. Phase I and Phase II trials including multiple tumor types are currently underway (Table 1).

#### 2.1.7. PF-04449913 (Glasdegib)

Glasdegib is a newly marketed product from Pfizer that has been shown to bind and inhibit SMO [100]. Studies have shown that glasdegib abrogates leukemia-initiation potential and leukemia stem cell dormancy in chronic myeloid leukemia (CML) and acute myeloid leukemia (AML) cells, most likely due to downstream GLI2 inhibition [101,102]. Several Phase II trials for treating hematological malignancies are currently underway (Table 1).

#### 2.1.8. PF-5274857

Pfizer concurrently discovered PF-5274857 to be a selective and potent SMO antagonist with the ability to penetrate the blood-brain barrier. *In vitro*, PF-5274857 was able to specifically bind to SMO and reduce *GLI1* gene expression in MEF cells [103]. The ability of PF-5274857 to penetrate the blood-brain barrier made it a prime candidate for treatment of brain tumors and brain metastases driven by aberrant HH signaling. In PTCH^+/−^ p53^+/−^ medulloblastoma allograft mouse models and primary medulloblastoma mice, PF-5274857 inhibited tumor growth and reduced *GLI1*, *GLI2*, *PTCH1*, and *PTCH2* gene expression with no significant side effects [103]. This makes it an ideal candidate for clinical trials in brain malignancies. No clinical trials to verify the effectiveness of PF-5274857 in humans are currently underway.

#### 2.1.9. TAK-441

Ohashi *et al.* first reported TAK-441 as a highly potent and orally bioavailable SMO inhibitor in 2012. In NIH3T3 cells transfected with GLI-luciferase reporter construct, reporter activity was significantly reduced in response to TAK-441 treatment [104]. TAK-441 inhibited growth in PTCH^+/−^ p53^+/−^ medulloblastoma allografts models in mice and was shown to significantly reduce *GLI1* mRNA expression in PAN-04 pancreatic tumor xenografts in mice [104,105]. Studies have also shown that in LNCaP prostate cancer xenografts mouse models, TAK-441 delayed castration-resistant progression of the disease and significantly suppressed *GLI1*, *GLI2*, and *PTCH1* gene expression. Upon androgen starvation, LNCaP cells, an androgen-sensitive prostate cancer cell line, upregulate Shh expression, but are normally insensitive to SMO antagonists. These data suggest that TAK-441 was able to inhibit the Shh pathway by suppressing Shh paracrine signaling [106]. TAK-441 has also been suggested to be effective in inhibiting Vismodegib-resistant SMO D473H mutant. In several different assays, TAK-441 was shown to have equal affinity for wild-type and mutated SMO, while Vismodegib and cyclopamine showed reduced affinity for the D473H mutant suggesting that TAK-441 may be clinically relevant to treating Vismodegib-resistant, Shh pathway-driven cancers [107]. There are no currently active clinical trials looking at treating any type of cancer with TAK-441.

#### 2.1.10. LY2940680 (Taladegib)

Taladegib is an experimental SMO antagonist developed by Lilly USA that binds directly to SMO and potently inhibits HH signaling in DAOY and C3H10T1/2 cells [108]. Wang *et al.* found that taladegib binds to the extracellular end of the transmembrane-helix bundle of SMO, inhibiting propagation of HH signaling [109]. In PTCH^+/−^ p53^−/−^ transgenic mice, taladegib reduced proliferation of spontaneously developed medulloblastoma and induced Caspase-3 activity signifying increased apoptosis. Taladegib also suppressed HH-mediated gene expression in subcutaneous xenograft tumor stroma and potently inhibited tumor growth. Importantly, taladegib was also shown to inhibit the activity of vismodegib-resistant SMO mutant (D473H) emphasizing its clinical potential [108]. Currently, taladegib is being tested in Phase I and Phase II trials for advanced solid tumors and esophageal cancers (Table 1).

#### 2.1.11. MRT-92

MRT-92, developed by Hoch *et al.* at the Neuroscience Paris-Saclay Institute in the Centre National de la Recherche Scientifique, was very recently reported to have anti-SMO activity by blocking several overlapping sites of the SMO transmembrane domain [110]. It displayed subnanomolar antagonistic activity against SMO, blocking SAG (SMO agonist)-induced trafficking of SMO at the primary cilium and SAG-induced differentiation of C3H10T1/2 cells. Hoch *et al.* also found that MRT-92 maintained similar pharmacological characteristics when binding to vismodegib-resistant SMO mutant [110]. Together these data indicate that MRT-92 is a very strong candidate for clinical trials, as its binding site overlaps those of many previous generation SMO antagonists, allowing for stronger binding affinity at a lower drug concentration. MRT-92 is not currently being tested in any clinical trials.

#### 2.1.12. Jervine

Jervine is a natural alkaloid isolated from the corn lily *Veratrum californicum*. It is a teratogen that inhibits the Shh-mediated response of chick neural plate cells and has also been shown to inhibit the growth of liver HepG2 and human colon carcinoma HT29 cell lines [67,111]. Jervine is able to inhibit HH signaling by binding SMO and preventing its conversion to an active state, leading to the accumulation of inactivated SMO in the primary cilium [112]. Clinical applications of jervine were never pursued due to its teratogenic side effects.

### 2.2. GLI Inhibitors

GLI transcription factors are the terminal effectors of the Shh-SMO signaling pathway and can also be activated independent of Shh and SMO by other important molecular pathways. Activation of GLI1 and GLI12 leads to upregulation of many pro-proliferative, pro-survival and pro-angiogenic genes, leading to tumor growth and therapeutic resistance [113]. Here we discuss inhibitors specifically targeting GLI transcription factors.

#### 2.2.1. GANTs

GLI antagonists, or GANTs, were discovered at the National Cancer Institute in a GLI-luciferase reporter assay screen in HEK293 cells [114]. GANT-58 and GANT-61 were both discovered to inhibit GLI-mediated gene activation, though GANT-61 showed more specificity towards GLI proteins and more effectively reduced GLI1 and GLI2 DNA-binding ability. GANT-61 has shown potent inhibition of GLI1 and GLI2 in many cancer cell lines, including rhabdomyosarcoma, osteosarcoma, neuroblastoma, and ovarian cancer [115,116,117,118]. In a human prostate cancer xenograft model in mice, GANT-61 reduced tumor growth and proliferation and strongly reduced expression of *PTCH1* mRNA [114]. No clinical trials are currently ongoing using GANT-61 to treat any type of cancer.

#### 2.2.2. Arsenic Trioxide (ATO)

Arsenic trioxide is an FDA-approved inhibitor of GLI1 and GLI2 transcription factors. It has been approved for treatment of acute promyelocytic leukemia [119]. ATO directly binds to GLI1 and GLI2, inhibiting activity and decreasing expression of canonical Shh-GLI genes [119,120]. Kim *et al.* found that ATO reduces the stability of GLI2 transcription factor, preventing its accumulation in the primary cilium in response to Shh signaling [121]. ATO has also been shown to increase apoptosis, reduce tumor cell growth, and decrease expression of Shh target genes *in vitro* and *in vivo* in osteosarcoma, acute promyelocytic leukemia, malignant pleural mesothelioma, malignant rhabdosarcoma, prostate, and colon cancer cell lines and xenograft models [122,123,124,125,126,127]. Studies have shown that ATO also reduced the viability of pancreatic cancer stem cells and prostate cancer-initiating cells, underlining its effectiveness in killing of tumor epithelial cells and tumor-initiating cells [122,128]. Arsenic trioxide is currently in several clinical trials ranging from Phase I to Phase IV for both solid tumors and hematological malignancies (Table 1).

### 2.3. Shh Inhibitors

SHH is the most potent of the three Hedgehog ligands [10]. Consequently, targeting Shh has come under consideration to inhibit cancers with dysregulated Shh pathway activation. While these therapeutic approaches have not reached the clinic, they have been shown to successfully inhibit the Shh pathway.

#### 2.3.1. RU-SKI 43

Following translation and synthesis of the Shh protein, the signal peptide is cleaved resulting in a 19-kDa product. During the final steps of SHH synthesis, the enzyme SHHat, a membrane-bound *O*-acyltransferase, catalyzes the attachment of palmitate to SHH [129,130]. Palmitoylation is critical to the potency of Shh as SHHat knockout mice showed developmental defects that mirrored Shh knockout mice [131]. As such, SHHat has become a target to inhibit the efficacy of SHH signaling. A screen for inhibitors of SHHat has resulted in RU-SKI 43, which inhibits SHHat activity and consequently inhibits Shh signaling in cultured cells [132]. Knockdown of SHHat or exposure to RU-SKI 43 reduced proliferation and anchorage-independent growth of breast cancer cells [133]. This approach was further shown to inhibit pancreatic tumor growth in animal models [134]. This approach has significant promise but has not yet reached human trials.

#### 2.3.2. Shh Monoclonal Antibody 5E1

Inhibition of Shh with antibody targeting has also been attempted. The Shh monoclonal antibody 5E1 has been shown to inhibit growth of medulloblastoma in mouse models [135]. Furthermore, animals receiving 5E1 shown a reduced tumor proliferation index, increased tumor cell apoptosis, and had better survival compared to cyclopamine-treated mice [135]. The 5E1 antibody has also been shown to reduce growth of pancreatic tumors in mice [136]. Targeting Shh via monoclonal antibodies has also not reached human trials.

### 2.4. Therapeutic Targets Regulated by tGLI1

Clinical efficacy of SMO-targeted therapies has been mixed while GLI-targeted treatments are still in preclinical testing. Clearly, there is a need to deepen the biological understanding of tumors with aberrant SMO and GLI activities. To this end, our lab identified a truncated, gain-of-function isoform of the GLI1 transcription factor, tGLI1, that is only present in cancerous cells and is undetectable in normal cells [62], making it an ideal drug target. We have shown that tGLI1 is a gain-of-function GLI1 that has a higher propensity than GLI1 to induce aggressive cancer phenotypes in both breast cancer and glioblastoma, including, growth, invasion, migration, and angiogenesis [54,62,63,64,65]. These observations point to the need to pharmacologically target tGLI1.

Currently, there is no means to specifically inhibit tGLI1. However, several tGLI1 target genes can be targeted using already developed agents, including, CD24, VEGF-A, VEGFR2, HPA1 and TEM7. Studies have attempted to target CD24 with a monoclonal antibody (mAb) in several cancer models, including lung, ovarian, colorectal, and pancreatic cancer [137,138]. These studies showed decreased proliferation, motility, and tumorigenicity of cancer cell lines in mouse models, and one study showed specifically that combination therapy of CD24 mAb and gemcitabine strongly potentiated its anti-cancer efficacy in a mouse lung cancer model [138]. Several steps towards marketing a CD24 mAb as a cancer therapeutic have been made. A Phase I clinical trial (NCT02650895) studying the safety profile of a CD24 mAb in healthy adults was recently completed, though no results have been published, and a patent was filed for the use of a CD24 inhibitor to treat neoplastic conditions [139]. CD24 remains a viable target for targeting the HH-tGLI1 pathway and further refinement of immunotherapies could prove to be beneficial in treating tumors with tGLI1 expression.

Antiangiogenic therapies have been established as a new focus for cancer drug development, as increased tumor vascularization is indicative of aggressive cancer phenotypes and required for growth and metastasis [140]. Heparanase, also recognized as HPSE or HPA1, is another tGLI1 target gene that has been more extensively researched as an antiangiogenic, anti-cancer drug target [141]. Several classes of heparanase inhibitors have been studied, the most characterized of which include the heparan sulfate mimetics PI-88 and PG545. Researchers have shown in a myriad of cancer models that PI-88 and PG545 potently inhibit tumor angiogenesis by binding the active site of heparanase, and can be used in combination with other chemotherapeutics or as an adjuvant therapy [142,143,144,145]. PI-88 has been tested in multiple Phase I, II, and II trials spanning solid tumors and hematological malignancies, while PG545 is currently being tested in a Phase I clinical trial for advanced solid tumors (NCT02042781). PI-88 was fast-tracked for FDA approval in 2007 for treatment of post-resection hepatocellular carcinoma. Roneparstat, a heparin mimetic, has also been shown to have strong anti-cancer therapeutic effects and is currently in a Phase I trial for multiple myeloma (NCT01764880) [146].

TEM7, also termed PLXDC1, has been used as a prognostic marker for resectable gastric and colorectal cancers. As currently understood, TEM7 does not appear to be a viable therapeutic target, though it retains usefulness as a key prognostic marker for progression, invasion, and metastasis in several types of solid tumors [147,148]. There are no clinical trials currently testing experimental therapeutics targeting TEM7.

VEGF-A and VEGFRs are the most widely characterized targets for antiangiogenic therapeutics and these drugs have shown a varying range of effectiveness across tumor types [149]. There are currently two FDA-approved immunotherapies for targeting VEGF-A and VEGFR interactions. Bevacuzimab, trade name Avastin, is a humanized monoclonal antibody that inhibits VEGF-A by direct binding [150]. It is currently approved as either first-line monotherapy or in combination for metastatic colorectal cancer, non-small cell lung cancer, metastatic renal cell carcinoma, glioblastoma, and a variety of other advanced solid tumors [151]. In 2011, the FDA revoked its approval for the use of bevacuzimab in treating breast cancer, citing a lack of advantage in survival rates, no improvement in quality of life, and significant side effects [152]. Currently, over 500 active clinical trials are using bevacuzimab, both as a monotherapy and in combination, to treat a variety of solid tumors. Ziv-aflibercept, trade name ZALTRAP, is the other FDA-approved immunotherapy for targeting VEGF-A, though its mechanism of action is slightly different from bevacuzimab. ZALTRAP is a recombinant fusion protein that acts as a decoy VEGF receptor, binding to VEGF-A and preventing it from interacting with VEGFR-1 and VEGFR-2 [153]. It was approved by the FDA in 2012 for use in combination therapy with several other chemotherapeutics for treatment of metastatic colorectal cancer that has progressed following oxaliplatin treatment [154]. There are currently a multitude of active clinical trials testing ZALTRAP in a variety of solid tumors. 

Our laboratory has shown that in addition to increasing VEGF-A expression, tGLI1 also increases expression of VEGF-C. The library of compounds for targeting VEGF-C is not as extensively developed as the one for VEGF-A, although there is one immunotherapy showing promising results in clinical trials. Circadian Technologies created a human monoclonal antibody named AGX-100 that is currently undergoing a first-in-human Phase I clinical trial both as a monotherapy and in combination with bevacuzimab for the treatment of metastatic solid tumors (NCT01514123) [155].

We have shown that tGLI1 is also able to upregulate transcription of VEGFR2, creating a powerful autocrine loop with VEGF-A [65]. VEGFR2 has long been a key target in the development of antiangiogenesis drugs as binding of VEGF-A to VEGFR2 accounts for the majority of the pro-angiogenic signals as observed in mouse models [156]. There are currently nine FDA-approved drugs for targeting VEGFR2, eight of which show multi-kinase inhibitory activity (Table 2).

In addition to targeting tGLI1 downstream target genes, inhibiting tGLI1 synthesis and targeting its transcription co-regulators could also inhibit tGLI1 leading to tumor cell kill. Unfortunately, the splicing events leading to tGLI1 synthesis are still unknown. Also unknown is whether tGLI1 requires transcription co-factors for its gain-of-function transcriptional functions. GLI1 has been shown to cooperate with transcription co-factors to activate target gene expression, including SMAD4, PCAF, and SAP18 [157,158]. Furthermore, whether tGLI1 is subjected to regulation by non-canonical pathways has not been investigated. Ideally, tGLI1 activity can be blocked by inhibiting the non-classical pathways that activate tGLI1 activity. Filling these knowledge gaps of tGLI1 will help with developing strategies to target tGLI1-driven tumors.

**Table 2:** FDA-approved inhibitors targeting VEGFR2.

Name	Company	Molecular Target(s)	Clinical Indications
Sorafenib (Nexavar)	Bayer Onyx Pharmaceuticals	VEGFR1-3 [159] PDGFRβ Raf	Unresectable Hepatocellular Carcinoma [160] Renal Cell Carcinoma [161] Radiation-Resistant Thyroid Cancer [162]
Sunitinib (Sutent)	Pfizer	VEGFR2 [163] PDGFRβ [164] Flt3 [164]	Renal Cell Carcinoma [165] Imatinib-Resistant Gastrointestinal Stroma Tumors [166]
Pazopanib (Votrient)	GlaxoSmithKline	VEGFR1-3 [167] PDGFR FGFR1-3 EGFR c-Kit	Metastatic Renal Cell Carcinoma [168] Metastatic Soft Tissue Sarcoma [169]
Regorafenib (Stivarga)	Bayer	VEGFR1-3 [170] PDGFRβ FGFR-1 KIT RET B-Raf	Metastatic Colorectal Cancer [171] Metastatic Gastrointestinal Stromal Tumors [172]
Vandetanib (Caprelsa)	Genzyme	VEGFR2-3 [173] EGFR RET	Metastatic Medullary Thyroid Cancer [174]
Cabozantinib (Cometriq)	Exelixis	VEGFR2 [175] c-MET RET	Medullary Thyroid Cancer [176]
Lenvatinib (Lenvima)	Eisai Inc	VEGFR1-3 [177] FGFR1-4 PDGFRα c-Kit RET	Radiation-Resistant Differentiated Thyroid Cancer [178]
Axitinib(Inlyta)	Pfizer	VEGFR1-3 [179] PDGFRα,β c-Kit	Renal Cell Carcinoma [180]
Ramucirumab (Cyramza)	Eli Lilly	VEGFR2 [181]	Gastric Adenocarcinoma [182] Gastro-Esophageal Junction Adenocarcinoma [182] Metastatic Non-Small-Cell Lung Cancer [183]

## 3. Conclusions

The Shh signaling pathway is highly complex that has been shown to play important roles in promoting oncogenesis, tumor growth and progression, and tumor drug resistance. Therefore, several components of the Shh pathway (Shh, SMO, and GLI1/2) are viable therapeutic targets for anti-cancer therapies. Since SMO and GLI1/2 can be activated by Shh-independent stimuli, targeting them potentially target the transduction of a number of important molecular pathways. Single agent SMO inhibitors has already been found to be relatively effective in treating some malignancies in preclinical experiments and two of these orally active agents have received FDA approval for use in treating advanced or metastatic BCC. The sheer number of clinical trials using SMO inhibitors highlights the importance of pharmacological targeting of SMO in cancer. However, these inhibitors also showed limited efficacy against a number of cancers. Therefore, further advances must also be made to understand the mechanisms of resistance to small molecule SMO inhibitors and how to overcome them. Case reports from medulloblastoma patients treated with vismodegib indicate two specific mutations that occur in response to treatment, both presenting on the target SMO [69]. The SMO G497W mutation results in a partial blocking of the drug entry site while the SMO D437H mutation totally disrupts hydrogen bond stability in the binding site [184]. Further development of drugs such as MRT-92, which binds multiple sites on SMO and shows activity against the SMO D473H mutant, is crucial to overcoming treatment-induced resistance [110]. Given the facts that tGLI1 is expressed in a tumor-specific fashion and behaves as a more potent transcription regulator compared to GLI1, an important task is warranted to target tGLI1-driven malignancies directly or indirectly by targeting its upstream regulators and downstream targets. 
